# Colorectal Cancer Diagnostic Methods: The Present and Future

**DOI:** 10.7759/cureus.37622

**Published:** 2023-04-15

**Authors:** Sai Sravya Gude, Rithik S Veeravalli, Bhuvanasai Vejandla, Sai Sreeya Gude, Tejaswi Venigalla, Venkateswarlu Chintagumpala

**Affiliations:** 1 Oncology, University of Houston, Houston, USA; 2 Medicine, University of Cincinnati College of Medicine, Cincinnati, USA; 3 Emergency Medicine, Loyola University Chicago, Chicago, USA; 4 Internal Medicine, Guntur Medical College, Guntur, IND; 5 Internal Medicine, Einstein Medical Center Montgomery, East Norriton, USA; 6 Internal Medicine, Crozer-Chester Medical Center, Upland, USA

**Keywords:** screening colonoscopy, colo-rectal carcinoma, flexible sigmoidoscopy, presence of blood in stool, serum biomarkers, artificial intelligence in medicine, colon cancer prevention

## Abstract

To meet the needs of the colorectal cancer (CRC) patient population, colorectal cancer screening is continuously updated. The most significant advice is to start CRC screening exams at age 45 for people at average risk for CRC. CRC testing is divided into two categories: stool-based tests and visual inspections. High-sensitivity guaiac-based fecal occult blood testing, fecal immunochemical testing, and multitarget stool DNA testing are stool-based assays. Colon capsule endoscopy and flexible sigmoidoscopy are visualization examinations. There have been arguments about the importance of these tests in detecting and managing precursor lesions because of the lack of validation of screening results. Recent advancements in artificial intelligence and genetics have prompted the creation of newer diagnostic tests, which require validation in diverse populations and cohorts. In this article, we have discussed the present and emerging diagnostic tests.

## Introduction and background

Introduction

Colorectal cancer (CRC) is a globally prevalent healthcare problem. It is the third most prevalent cancer in men and women and the second leading cause of cancer death worldwide [1,2]. With such a widespread disease, early detection for effective treatments is essential in reducing the mortality rate of the disease. An emphasis has been placed on creating standards for these guidelines and ensuring that the populations most at risk are at the focal point [3]. Incidentally, as the global occurrence of CRC nearly doubled from 1990 to 2013, the general trend in mortality rate over the same timeline has decreased [1]. This article reviews the recent changes and updates in CRC screening guidelines and details what tests are available now and what types of tests may be available in the future.

Screening Guidelines

The American Cancer Society (ACS), US Preventative Services Task Force (USPSTF), and US Multi-Society Task Force are three prominent American organizations that oversee CRC screening recommendations [4]. The organizations create the guidelines based on extensive reviews of accumulated data and trends, and the recommendations they make influence the decisions made at a legislative level [4].

In 2018 the ACS provided a guideline update for CRC screening. The suggestions were categorized as either solid or qualified recommendations. The ACS advises that persons aged 45 and older with an average risk of colorectal cancer undergo routine screening using either a high-sensitivity stool-based test or a structural examination [5]. They issued a qualified recommendation to begin annual CRC screenings at the age of 45 and a strong recommendation for annual CRC screening in those 50 years and older [5]. The stool-based tests highlighted in their guidelines were the fecal immunochemical test (FIT) test, the guaiac-based fecal occult blood test (gFOBT), and the multitarget stool DNA test. The structural examinations highlighted were the colonoscopy, computerized tomography colonography examination, and flexible sigmoidoscopy [5].

In 2021 the USPSTF issued an updated recommendation statement for CRC screening. The recommendations assessed the benefits of screening at different age intervals and classified them by substantial and moderate levels. The USPSTF found substantial benefits in regular CRC screening for people aged 50 through 75 years and moderate benefits in regular CRC screening for those aged 45 through 49 years [6]. These conclusions were made in reference to high-sensitivity stool-based tests and structural examinations. For those average-risk populations, the USPSTF recommends a high-sensitivity gFOBT and FIT test every year and a multitarget stool DNA test every one to three years. In addition, they recommend a colonoscopy every ten years and CT colonography and flexible sigmoidoscopy examinations every five years [6].

Lastly, the USPSTF also updated its CRC screening guidelines in 2021. The American Gastroenterological Association, the American Society for Gastrointestinal Endoscopy, and the American College of Gastroenterology (ACG) work alongside the USPSTF to revise and create screening guidelines. Strong recommendations and conditional recommendations categorized the new guidelines. They strongly recommended CRC screening in average-risk populations of those between the ages of 50 and 75 and conditionally recommended CRC screening in those average-risk populations for those between the ages of 45 and 49 [7]. The new guidelines also categorized the recommended screening tests into three distinct levels: first tier, second tier, and third tier. Tier one tests are considered the most crucial and practical and include the annual fecal immunochemical test (FIT) test and the colonoscopy exam every 10 years. Tier two tests included CT colonography and flexible sigmoidoscopy every five years. The tier three tests include the capsule colonoscopy every five years [7].

## Review

Current CRC screening guidelines

Fecal Occult Blood Test (FOBT)

The principle behind the fecal occult blood test (FOBT), used to diagnose CRC clinically and screening, is that CRCs leak tiny traces of occult blood (macroscopically invisible) into the gut lumen. FOBT tests analyze stool samples for this in occult blood [8]. Currently, there are two types of FOBT tests readily available and being used. They are guaiac-based tests (gFOBTs) and immunochemical tests (iFOBTs). 

Guaiac-based tests utilize a chemical reaction based on the pseudo-peroxidase activity in hemoglobin; it can identify hidden blood in the stool. This test involves taking a sample of the patient's stool and smearing it onto guaiac paper. If the stool contains hemoglobin, it will turn blue due to an oxidative reaction [4]. Currently, gFOBT is the most common form of CRC screening due to its simple nature, availability, and cost. Since FOBT is a nonspecific test for gastrointestinal bleeding, it has a relatively poor sensitivity for CRC, which results in false positives for other gastrointestinal bleeding (erosions and/or ulcers, inflammatory bowel disease, or medication with antiplatelet agents) [8]. Furthermore, dietetic provisions are necessary to exclude false-positive results. A recent study showed limited sensitivity to this test for advanced adenomas (11%) and carcinomas (12%). With the use of gFOBT, a decrease in mortality for CRC by 15-33% has been proved [9].

Immunochemical Fecal Occult Blood Test (FIT)

The immunochemical fecal occult blood test, which uses human hemoglobin, particularly for detection, has a higher sensitivity than gFOBT and a similar specificity to gFOBT for detecting advanced neoplasia [10]. IFOBT is the other type of FOBT that is readily available. To identify human hemoglobin, FIT uses specific antibodies. The test is based on the idea that an appropriate monoclonal or polyclonal antibody can recognize and bind to the intact globin component of human hemoglobin [11]. Compared to FOBTs, fecal immunochemical tests (FITs) are more sensitive to detecting CRC and adenomas. The ease of use of FITs is increased because they call for one or two stool samples, and there are no dietary or drug limitations [12]. 

In terms of participation and positive rate, FIT performed better. Additionally, the number need to screen (NN-screen; 84% vs. 31-49% at different cut-off levels) and number need to scope (NN-scope; 2.2% vs. 1.6%), as well as false positive and false negative rates, exhibited significant differences in FOBT vs. FIT, respectively (RR:-4.06; 95% CI -7.89-0.24) [10]. The only downside to FIT is that it costs significantly more to run than gFOBTs [8]. Even with that downside, in many other nations, including the US, FIT has primarily taken the place of high-sensitivity FOBT (hsFOBT) and gFOBT [4].

Multitarget Stool DNA Testing 

Regarding the test, the multitarget stool DNA test (mt-sDNA) is similar to FIT. The multitarget stool DNA test includes immunochemical assays for human hemoglobin and molecular assays for abnormally methylated bone morphogenetic protein 3 (BMP3) and N-Myc downstream-regulated gene 4 (NDRG4) promoter regions, mutant Kirsten rat sarcoma (KRAS) virus, and -actin. A validated, predetermined logistic regression technique was used to combine the quantitative reading of each marker; a value of 183 or more invalidated a positive test result [13].

Compared to FIT, mt-sDNA produces more false positive results. Additionally, it was found that neither mt-sDNA nor FIT had significantly higher sensitivity for detecting advanced adenomas with a high risk of progression [4]. That said, 56 of the 60 people with screening-relevant malignancies were identified by multitarget stool DNA testing, with sensitivities of 92.3% and 93.3, respectively [14].

Computed Tomography Colonography 

CT Colonography (CTC) is a developing non-invasive imaging method that depends on the execution of a thin-section colon CT scan and data analysis using two- and three-dimensional images [15]. For the procedure to be successful, cathartic bowel preparation and air insufflation for colonic distension to the highest level tolerated (about two liters of room air or carbon dioxide) are required [16]. In more recent times, patients have been given oral contrast agents. 

A significant, multicenter US Department of Defense trial comparing colonoscopy and CTC in 2003 revealed that the latter had a 96% specificity and 94% sensitivity for large (>1 cm) adenomas. Using a smaller size threshold (6mm or greater), the sensitivity and specificity of CTC dropped from 89% to 80%. Additionally, CTC examination results are very dependent on the radiologist rather than gastroenterologists. Therefore, it is essential for analysis techniques to stay consistent across the board. CTC does come with its disadvantages, though. A major one, in fact, is the significant exposure to radiation a patient endures during the procedure [16].

Colon Capsule Endoscopy

Colon capsule endoscopy (CCE) is a safe and effective tool for detecting CRC and polyps in a screening setting [17]. The CCE uses a disposable capsule that passes through the colon using peristalsis to record color video footage from both ends. The device's two cameras allow for 344-degree coverage of the intestinal mucosa [17]. CCE is available in two generations, with the latest generation exhibiting improved polyp detection [4]. In 2006, colon capsule endoscopy was first introduced by Eliakimetal. First-generation colon capsule endoscopy had a moderate sensitivity for detecting polyps of more than 6 mm. Thus, second-generation colon capsule endoscopy was developed for higher sensitivity [18]. The majority of CCE research has concentrated on high-risk patients with hsFOBT or FIT readings that are positive or who have symptoms suggestive of CRC. Based on the scant evidence supporting its use in a population at average risk for screening, the USPSTF guidelines classify CCE as a tier three CRC screening test. CCE is listed as an alternative in the American College of Gastroenterology screening recommendations for people who cannot have a colonoscopy or FIT. The US Food and Drug Administration has not yet approved CCE for routine CRC screening in individuals with average risk, despite the fact that it is permitted to inspect the colon in patients who are at high risk for complications from colonoscopies [4].

Flexible Sigmoidoscopy 

Flexible sigmoidoscopy (FS) is an endoscopic CRC screening modality that allows for direct visualization of the rectum, sigmoid, and descending colon [19]. Direct colonic viewing, tissue sample, and polyp removal are all made possible by FS, but only on the left side of the colon. At 11 years of follow-up, FS was proven to lower CRC incidence by 20% and CRC death by 27%. Despite the fact that FS has been shown to be effective as a CRC screening test, it is underutilized in the US, in part because colonoscopy studies have shown superior findings and the test is non-sedated [4]. The resources required for flexible sigmoidoscopy are similar to a colonoscopy. Still, a colonoscopy is needed to follow up on a positive FIT and for those with polyps on flexible sigmoidoscopy. The lack of sedation and fear of pain are barriers to participation in flexible sigmoidoscopy [2].

Colonoscopy

Colonoscopy has been utilized in various studies of hsFOBT, FIT, mt-sDNA, and other non-colonoscopic test modalities as the gold standard comparative test. Although invasive, colonoscopy enables visibility, sampling, and removal of malignant or precancerous tumors and longer gaps between screenings; after a regular examination, a colonoscopy is advised every 10 years. Furthermore, a colonoscopy can assess the lesion's features, such as differentiating between an adenoma and a hyperplastic polyp or determining whether a lesion is precancerous or in the early stages of malignancy [4]. Additionally, a colonoscopy provides the added benefit of enabling therapeutic measures like biopsies, polypectomy, or the removal of early-stage cancer while also preventing fatalities from CRC [20]. 

It has been demonstrated that screening colonoscopies can lower the incidence of adverse CRC outcomes [4]. Colonoscopy screening is not a cheap or easy test that can be easily applied to the at-risk population. If a colonoscopy is available, a patient who has tested positive for other screening tests such as the FOBT, sigmoidoscopy, or computed-tomographic colonography should be referred to. Direct colonoscopy appears to be the most common method of CRC screening, despite the fact that resources are easily accessible for screening in many nations. A colonoscopy often results in 1-2 complications per 1000 procedures (World Gastroenterology Organization, 2021) [21].

Methylated Septin 9 

The guanosine triphosphate protein (GTP)-binding protein Septin 9 (SEPT9) is a member of the GTP family, and SEPT9 methylation is connected to carcinogenesis and acts as a biomarker for CRC [4]. The SEPT9 V2 gene transcription's gamma promoter region, which is differentially methylated in patients with CRC, is analyzed as part of the Septin 9 test, which has been shown to be effective in the non-invasive and accurate screening of CRC. It is a potential blood-based biomarker for diagnosing CRC [22]. It is a non-invasive, patient-friendly test with reasonable compliance expectations [23]. Its efficacy as a screening test for average-risk individuals without symptoms has been questioned. The USPSTF recommendations warn against utilizing methylated Septin 9 (mSEPT9) for this reason since it is more likely to detect advanced-stage neoplasia than CRC. Studies have shown that mSEPT9 has a sensitivity of 48% for CRC detection and 11% for advanced adenoma detection [4].

Emerging colorectal cancer screening tests 

Numerous significant and encouraging developments have taken place in CRC screening in the last ten years, such as the development of novel screening procedures and the improvement of current technology to detect colonic neoplasia better [4]. In summary, to remain a relevant participant in CRC screening, it is important to understand the various screening options' strengths and weaknesses, including those just now emerging [24].

Artificial Intelligence in CRC Screening

It is unsurprising that diagnostic tools for identifying cancer have been gradually integrating approaches to better diagnose patients with increased accuracy and precision as more technologies develop based on AI. Its capacity for handling vast amounts of data and its ability to extract information that experts cannot notice are further advantages. The effort to enhance medical imaging methods has started by looking at how deep learning can speed up the process of using imaging to find cancer. Other tools that can improve medical imaging methods include those that improve image quality by integrating 3D technologies into image extraction [25].

Although the introduction of AI into imaging modalities appears to be a natural fit, the potential use for assisting in the detection of genetic diseases and pathological disorders is equally promising and merits equal attention. This might necessitate modifications to how diseases are now diagnosed, or it might lead to the development of new diagnostic techniques. In general, improving current imaging or testing techniques can have revolutionary effects [25]. 

Important issues must be addressed, including developing a fundamental strategy for incorporating computer-aided diagnostics and determining how much we should rely on AI's support. Before accurate implementation becomes the norm, safety measures must be carefully studied [25]. At this moment, the most fundamental and initial step is the requirement for established AI medical ethical principles.

Micro RNAs (miRNAs) in Screening

Several of their distinctive qualities are the underlying causes of this growing interest. First off, micro RNAs (miRNAs) exhibit remarkable stability in a range of experimental and lab settings. Second, miRNAs are predicted because of their short size and hairpin-loop shape [26]. 

A single miRNA does not appear to be able to sufficiently capture the underlying disease heterogeneity in colorectal polyps and malignancies, despite the fact that an increasing number of miRNAs have been discovered as promising biomarkers for the early diagnosis of CRC. The combination of miRNAs into a biomarker panel has thus been suggested in a number of studies to increase the accuracy of the identification of colorectal neoplasms [26].

Histone Modifications as a Screening Tool

Studies have shown that this dysregulation most likely changes the gene expression patterns in CRC. It has been challenging to ascertain the histone modification status in primary cancer tissues, nonetheless, due to the technical constraints of assays that evaluate the post-translational histone modification state. As a result, research into whether histone modifications may be employed as disease biomarkers has been limited [26]. The development of CRC biomarkers has focused on global abnormalities of specific histones in primary tissues.

Capsule Endoscopy as a Screening Test

With regard to accessing the gut microbiota, little ingestible device modules hold out a lot of potential. In reality, swallowable, pill-sized capsules have only lately become a viable method for examining a number of other GI-related operations, such as imaging and endoscopy, core temperature, heart rate, pH, pressure, bleeding, and drug monitoring. Using ingestible devices has been extended to include monitoring biomarkers of interest in various digestive system segments [27].

The upper gastrointestinal system has only been used in clinical tests using this technology thus far (stomach and small intestine). The camera starts to lose power when it reaches the colon because the battery only lasts roughly eight hours. The slower fluid transit time in the colon compared to the small intestine further complicates the battery life issue. There is currently no evidence to support the use of capsule video endoscopy for the detection of colorectal polyps or cancers, and the manufacturer is not marketing it for that purpose [28]. However, there have been inquiries about using the device for colorectal cancer screening, as well as some sporadic commercial use. Equipment and technique must be improved, and the technology's capacity to identify advanced colorectal neoplasia must be assessed before it can be considered for diagnostic or screening use in the colon [28].

The Usefulness of Liquid Biopsy

The phrase "cell-free DNA" when referring to liquid biopsies refers to all DNA discovered in the plasma, of which circulating tumor DNA (ctDNA) is the subset only of cancer origin. CtDNA typically comprises 166 base pairs (bp) long short DNA fragments with an estimated half-life of 16 to 2.5 hours. Diagnostic molecular profiling of ctDNA appears to show high agreement with tissue-based assays in treatment-naive individuals with CRC [29].

Many epigenetic biomarkers, including DNA methylation, histone alterations, miRNAs, and long noncoding RNA, have shown promise as clinically significant biomarkers for CRC diagnosis, prognosis, and therapy response prediction [30].

The search for blood biomarkers of CRC has been conducted during the past ten years using a range of proteomics-based techniques. Biomarkers such as nuclear matrix proteins, C-type Cytochrome synthesis proteins CCSA-2, CCSA-3, CCSA-4, matrix metalloproteinase 9, S100A8, and S100A9 were identified through studies of the tumor tissue proteome. The relative ease of quantitation by immunoassay of these markers, despite the fact that their individual sensitivities and specificities in pilot studies fall short of acceptable performance for population screening, encourages their development as a component of an extended panel of serum protein biomarkers [31].

To assist clinicians in deciphering with high confidence and based on which we can identify that the patient doesn't have CRC, a non-invasive screening method with the best feasible rate of identifying true negatives is still required. Yet a screening tool like that has not been identified [32].

## Conclusions

Even though new treatments are available for primary and metastatic colorectal cancer and subsequent improvement in survival rates, screening rates have not appreciably increased. To improve the identification and prevention of this illness, finding more highly accurate, non-invasive, and tolerable CRC screening tests has become the need of the hour. In this article, we have discussed the current diagnostic methods and the possible emerging techniques.

The criteria for a better test of colorectal cancer screening can be divided into two categories -the patient's and clinician's perspectives. According to the patient's point of view, a serum test is better than a stool test. Most patients would rather avoid having a colonoscopy, which is highly intrusive and uncomfortable. The patient must handle their stool samples for the current FIT test before giving them back to the healthcare professional. The procedure could be upsetting for some patients, causing them to put off collecting and returning their samples. As a result, only about 50% of the population participates in screening. A serum blood test should be developed and launched to detect biomarkers as a result, as this is likely to be far more acceptable to patients and lead to greater uptake in the field. In the end, people want and demand a straightforward point-of-care test that, ideally, does not call for a stool sample and, if negative, can categorically exclude CRC. Results should be available quickly, particularly in cases when malignancy is suspected. From the physician's standpoint, the test's high specificity, ideally above 90%, is the most crucial requirement to be satisfied. If the test is negative, clinicians can rule out CRC safely. Another urgent need is to point patients toward a particular therapy or assess its effectiveness. Another crucial aspect of these tests is their cost-effectiveness. All of these tests would have to be created concurrently with the discovery of new chemotherapy treatments that can extend life and lower the possibility of recurrent illness.

Presently available and advised screening techniques have undergone extensive research. Therefore, governments and physicians must keep working to increase awareness and lower screening-related obstacles.
